# Improving Clozapine Prescribing at a London District General Hospital: A Quality Improvement Project

**DOI:** 10.1192/bjo.2024.351

**Published:** 2024-08-01

**Authors:** Jack Benson, Tara Amin, Diana Shroff, Kirsty Allen

**Affiliations:** 1 Barnet, Enfield and Haringey Mental Health NHS Trust, London, United Kingdom; 2 Royal Free Hospital NHS Trust, London, United Kingdom; 3 North Middlesex University Hospital NHS Trust, London, United Kingdom

## Abstract

**Aims:**

The liaison psychiatry team at North Middlesex Hospital (NMH) noticed that many patients on clozapine were missing doses in hospital, risking the need for re-titration and deterioration in mental state. Although clozapine is a widely used medication in psychiatry, non-psychiatric clinicians may not be aware of the importance of compliance. In addition, clozapine is often not widely available in acute medical hospitals and ascertaining the correct dosage can be difficult as it is not prescribed by the GP. Furthermore, clozapine can cause a variety of side effects that our medical colleagues may not be familiar with.

The aim of this project was to improve clozapine prescribing at NMH and improve communication with the liaison psychiatry team.

**Methods:**

We reviewed the notes of 97 admissions in which patients were dispensed clozapine from the hospital pharmacy during the period April 2020 to December 2023 to determine what proportion had missed a dose of clozapine, and the clinical implications of this. We also reviewed the reasons for the missed doses to gather information on what could be done to improve patient safety.

From July 2022 we began implementing changes. This included the creation of a hospital guideline, putting in place an automatic email that would be sent to the liaison team when clozapine was prescribed, placing an alert on the online prescribing system to emphasise the importance of not omitting doses, and providing teaching to clinicians.

**Results:**

We compared omissions of clozapine doses and referrals to the liaison team before and after changes were implemented. The percentage of patients inappropriately missing at least one dose fell from 67.4% to 31.1%. The proportion of patients who were referred to the liaison team rose from 40.8% to 89.2%.

We identified several recurring causes of missed doses. These included doctors not being aware of clozapine prescriptions or dosages, poor awareness that clozapine is a critical medicine and long stays in accident and emergency. There were also incidents where clozapine was stopped by the medical team without obtaining advice from psychiatric colleagues.

**Conclusion:**

We were able to reduce the proportion of patients missing doses by improving awareness of clozapine compliance within the hospital. We were also able to improve communication between medical and psychiatric teams.

The clozapine guideline and prescribing alerts will continue to be utilised within the hospital. We plan to continue to provide regular teaching to rotational junior doctors and to pursue a similar project for lithium prescribing.

